# Morphological Findings in Trophozoites during Amoebic Abscess Development in Misoprostol-Treated BALB/c Mice

**DOI:** 10.1155/2015/898467

**Published:** 2015-05-18

**Authors:** Andrés Aceves-Cano, Rocío Gaytán-Ochoa, Ernesto Ramos-Martínez, Gilberto Erosa de la Vega, Carmen González-Horta, Patricia Talamás-Rohana, Blanca Sánchez-Ramírez

**Affiliations:** ^1^Facultad de Ciencias Químicas, Universidad Autónoma de Chihuahua, Circuito No. 1, Nuevo Campus Universitario, 31125 Chihuahua, CHIH, Mexico; ^2^Departamento de Anatomía Patológica del Hospital CIMA, Avenida Hacienda del Valle No. 7120, 31217 Chihuahua, CHIH, Mexico; ^3^Departamento de Infectómica y Patogénesis Molecular, CINVESTAV-IPN, Avenida Instituto Politécnico Nacional 2508, 07360 Mexico, DF, Mexico

## Abstract

During amoebic liver abscess (ALA) formation in susceptible animals, immune response is regulated by prostaglandin E_2_ (PGE_2_) dependent mechanisms. The aim of this study was to analyze the effect of misoprostol (MPL), a PGE_1_ analogue, on ALA formation in BALB/c mice. Male mice from BALB/c strain were intrahepatically infected with 7.5 × 10^5^ trophozoites of *E. histolytica* strain HM1:IMSS and treated with 10^−4^ M of MPL daily until sacrifice at 2, 4, and 7 days postinfection (p.i.). ALA formation was evaluated at 2, 4, and 7 days postinfection; trophozoite morphology was analyzed using immunohistochemistry and image analysis. Results showed an increase in frequency of ALA formation in infected and MPL-treated mice only at 2 days p.i. (*P* = 0.03). A significant diminution in the size of trophozoites was detected in abscesses from mice independently of MPL treatment (from 5.8 ± 1.1 *µ*m at 2 days p.i. to 2.7 ± 1.9 *µ*m at 7 days p.i.) compared with trophozoites dimensions observed in susceptible hamsters (9.6 ± 2.7 *µ*m) (*P* < 0.01). These results suggest that MPL treatment may modify the adequate control of inflammatory process to allow the persistence of trophozoites in the liver; however, natural resistance mechanisms cannot be discarded.

## 1. Introduction

Amoebic liver abscess (ALA), produced by* Entamoeba histolytica *infection, is a common complication of invasive intestinal amoebiasis. Hamsters and gerbils are susceptible to hepatic amoebiasis [1, 2] while mice are resistant to infection either intracecally or intrahepatically [3]. Although basis for mice resistance is not totally known, several studies in susceptible animals have demonstrated the importance of macrophage function in host resistance during ALA formation [4]. Prostaglandin E_2_ (PGE_2_) is an important mediator of inflammation that can modulate T helper (TH) cells towards a preferential TH2 subgroup [5] and in this way regulate cooperator and effector macrophage functions.

The increase in PGE_2_ plasmatic levels and induction of the type 2 cyclooxygenase enzyme (COX-2) in infected liver, as well as the expression of COX-2 mRNA in macrophages and neutrophils, was reported by our group using the hamster, a susceptible experimental model [6, 7]. In these studies we found that the inhibition of PGE_2_ biosynthesis by indomethacin treatment had a beneficial effect limiting the inflammatory process [6]. Additionally, studies done in SCID mice in which a human intestinal xenograft was implanted have provided evidence that the infection with* E. histolytica* promotes an intense acute inflammatory reaction, accomplished by neutrophil infiltration with an increase in PGE_2_-COX-2 enzyme dependent levels [8]. These results suggest that PGE_2_ could be a host-dependent factor involved in susceptibility or resistance. Immunosuppressive therapy with PGE_2_ is limited by poor oral bioavailability and short half-life. However, several studies using the PGE_1_ analogue, misoprostol (MPL), have demonstrated that MPL shares, with PGE_2_ and PGE_1_, the ability to inhibit mitogenic activity of IL-1, tumor necrosis factor (TNF) (*α* and *β*), IFN-*γ*, IL-12, and IL-18 production [9, 10]. Moreover, MPL has been used to restore immune responsiveness and the expression of surface class II antigen and IL-2 receptors in lymphocytes [11, 12]. In this study we hypothesized that exogenous prostaglandin analogue (MPL) could induce, in a resistant model such as BALB/c mice, an increase in susceptibility. Results reported here demonstrate that MPL can modulate the inflammatory process to allow the persistence of trophozoites in the liver. Additionally, we report significant changes in trophozoites' morphology, which could be related with the mice natural resistance to* E. histolytica* infection.

## 2. Materials and Methods

### 2.1. Chemicals

Adult bovine serum was obtained from Equitech-Bio, (Ingram, TX, USA). Ethanol was obtained from Baker (Edo. de México, México); sodium pentobarbital (Anestesal*®*) was obtained from Pfizer S.A de C.V. (Toluca, Edo. De México, México). Gelfoam was obtained from Upjohn (Kalamazoo, MI, USA); suture 3/0 Curex was obtained from International Pharmaceutics (México); formalin and hematoxylin and eosin (H&E) were obtained from Merck (Darmstadt, Germany). For immunohistochemistry Histostain-plus kit (Zymed Lab. Inc., San Francisco, CA, USA). Misoprostol (MPL) was a gift from the G.D. Searle, Co. (México).

### 2.2. *Entamoeba histolytica* Cultures

*E. histolytica* strain HM1:IMSS was kindly provided by Dr. V. Tsutsumi (Center for Research and Advanced Studies, National Polytechnical Institute, México City, México) and passed three times through hamster liver to preserve virulence. The strain was maintained in our laboratory by subculturing twice a week in axenic TYI-S33 medium [13]. Cultures for inoculation were grown in 15 mL screw-capped tubes. Log-phase cultures were chilled on ice for 5 min. Trophozoites were pelleted by centrifugation at 500 ×g for 5 min in a Sorvall RT6000B refrigerated centrifuge, counted on a hemocytometer, and resuspended in the same medium to yield a cell density of 7.5 × 10^6^ cell/mL and 1.5 × 10^7^ cell/mL for inoculation in hamsters and mice, respectively. Sterile screw cap vials containing amoebas were kept on ice, pending inoculation.

### 2.3. Amebic Liver Abscess Development

Male inbred hamsters (*Mesocricetus auratus*) weighing approximately 100 g were used as controls for amoeba virulence, liver damage, and trophozoite morphology. BALB/c mice weighing approximately 30 g were divided in four groups: infected or not and with or without MPL treatment. Hamsters and mice were infected intrahepatically with 7.5 × 10^5^ trophozoites of* E. histolytica* in mid-log phase as described previously [6]. Briefly, animals were anaesthetized with sodium pentobarbital diluted 1 : 10 in phosphate buffer saline pH 7.4 and applied i.p. (1.5 mL/100 g of body weight). After a longitudinal midline incision, approximately 1.5 cm in length, the liver was exposed and the inoculum (0.1 mL for hamsters and 0.05 mL for mice) was slowly injected using a tuberculin syringe equipped with 29G × 13 mm needle. Gelfoam was used to prevent hemorrhages and was removed before suturing, using 3–0 normal suture. Aseptic precautions were observed throughout infection procedure and during postinfection time. At 2, 4, and 7 days postinfection (p.i.) animals were anaesthetized and killed by exsanguination, and livers and abscesses were dissected and weighted to determine the percentage of damage as the ratio between abscess and liver weight before abscess removal. Liver and abscesses were fixed with 3.8% phosphate-buffered formalin, and paraffin embedded. Paraffin sections were stained with hematoxylin and eosin (H&E) to confirm amoebic invasion.

### 2.4. Treatment

Treatment was used only in mice groups; for this, 2 mg of MPL was dissolved in 1 mL of ethanol and further diluted with sterile water to obtain a final concentration of 1 × 10^−4^ M of MPL. From this solution, 0.1 mL (128 *μ*g/100 g of body weight) was applied 1 h previous to infection in treated groups and daily until sacrifice. This dose was considered by other studies as immunosuppressive [9, 12]. A treated and noninfected group was included to discard morphologic features due to MPL effects in hepatic tissue. Consumption water for MPL-treated animals, infected or not, was added with 0.25 *μ*g/mL of MPL, to avoid variation in MPL plasmatic levels.

### 2.5. Immunohistochemical Analysis

Paraffin liver sections (6 *μ*m thick) were obtained from each group and processed for immunohistochemical (IHC) analysis to detect trophozoites. IHC was performed as described previously [7]. Briefly, paraffin sections (4 *μ*m thick) were deparaffinized with xylene, rehydrated in a graded series of ethanol, and equilibrated in phosphate-buffered saline bath for 5 min. Endogenous peroxidase quenching treatment was done by incubating samples for 10 min at room temperature in absolute methanol containing 1% hydrogen peroxide. Blocking step was performed following instructions of Histostain-plus kit. The primary antibody, a polyclonal rabbit anti-amoeba antiserum (1 : 500 dilution), was detected using the secondary affinity-purified biotinylated goat anti-rabbit IgG antibody, avidin-peroxidase, and freshly prepared diaminobenzidine substrate. All these reagents were used as provided by the manufacturer. Nonrelated or preimmune sera were used as negative controls.

### 2.6. Image Analysis and Trophozoites Measurements

Trophozoite measurements were done using a BX41 Olympus microscope equipped with a Pixera-CCD camera and analyzed with the IMAGE Pro Plus 4.1 software (Media Cibernetics, Silver Spring, Maryland, USA). The size of trophozoites present in ten microphotographs taken from damaged areas of each liver (40x) was measured as major (MD) and minor diameter (md) using the IMAGE software measure tool, previously calibrated. Microscope calibration was done using a stage micrometer (1 mm/100 parts; Euromex Microscopes Holland BV, Arnhem, Holland) with a value of 5.240 pixels/*μ*m. All determinations were made the same day to diminish calibration or lighting errors.

### 2.7. Statistical Analysis

Liver damage data were analyzed as quantitative variables using an ANOVA test; differences between groups were considered significant when *P* ≤ 0.05 by Wilcoxon analysis. Trophozoites' measurements data are presented as means ± standard deviation. The differences in trophozoites' dimension between groups and among species were analyzed by Student's *t*-test and considered significant when *P* < 0.05. Comparisons between experimental and control groups were performed using an analysis of variance (ANOVA), where appropriate. Data analyses were carried out with the STATA 9.0 program for Windows (Stata Statistical Software, Release 9.0., Stata Corporation, College Station, Texas, USA).

## 3. Results

Intrahepatic inoculation of* E. histolytica *trophozoites in hamsters induced ALA formation in 100% of the animals, which corroborated the virulence of the amoeba strain. A significant increase in liver weight was observed in infected hamsters in comparison to liver weight of noninfected hamsters; however, in the case of infected BALB/c mice groups, liver weight did not suffer significant changes neither in MPL-treated animals nor in untreated mice (Table 1). Liver damage in hamsters increased significantly until it reached 35% at 7 days p.i, while damage observed in BALB/c mice was less than 15% (Table 1) at different times p.i., and no significant difference was detected among infected mice due to MPL treatment. Although ALA formation was more frequently observed in MPL-treated and infected mice in comparison to untreated and infected mice, ALA development was significantly different only at two days p.i. (*P* < 0.05) compared with untreated and infected mice (Table 2).

**Figure 1:**
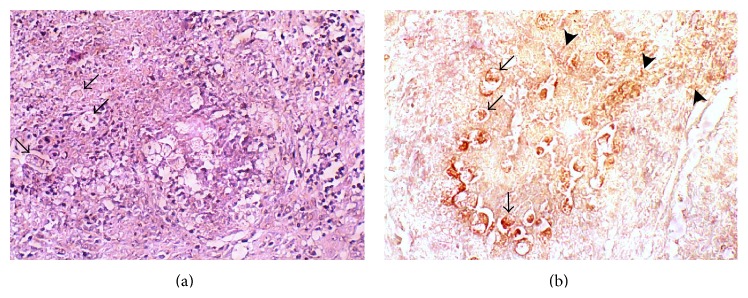
Microphotography of histological changes observed in liver from hamsters infected with* E. histolytica* at four days postinfection. (a) Numerous amoebic trophozoites (arrows) present in liver tissue. H&E stain; original magnification 10x. (b) Immune detection of trophozoites showing a strong peroxidase reaction (arrows); multiple signals of amoebic proteins were observed in areas adjacent to trophozoites (arrow heads). Original magnification 10x.

Abscesses in liver from infected hamsters had macroscopic characteristics similar to those described previously [1]. Microscopically, the hepatic parenchyma showed multiple foci of acute inflammatory reaction, enlarged granulomas, and intense necrosis areas. Cellular infiltrate was composed by polymorphonuclear leukocytes and eosinophils and histiocytes were present as an inner layer. Livers from infected hamsters at 2, 4, and 7 days p.i. showed the presence of numerous trophozoites mainly at the periphery of necrosis which was corroborated by IHC (Figure 1).

Livers from infected mice treated or not with MPL had normal surface and color; abscesses were grossly observed and frequently showed a grey-white coloration. To confirm ALA formation in mice, microscopic examination was performed to demonstrate foci of inflammatory infiltrate, trophozoites, and/or necrosis.

**Figure 2:**
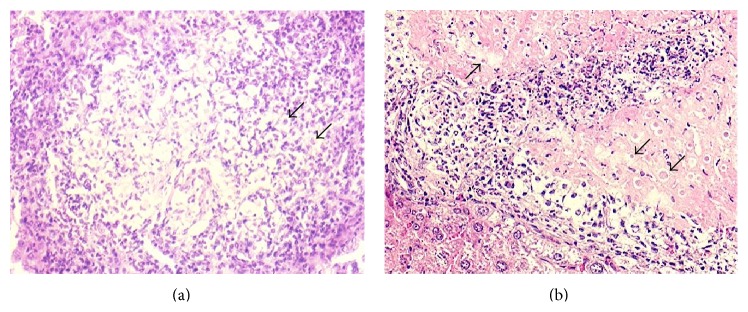
Effect of MPL treatment on ALA formation in BALB/c mice at two days postinfection. (a) Histological changes in mice infected without MPL treatment. Original magnification 10x. (b) Histological changes in infected mice and MPL-treated mice. Original magnification 10x. Arrows indicate the trophozoites in the hepatic tissue. H&E stain.

**Figure 3:**
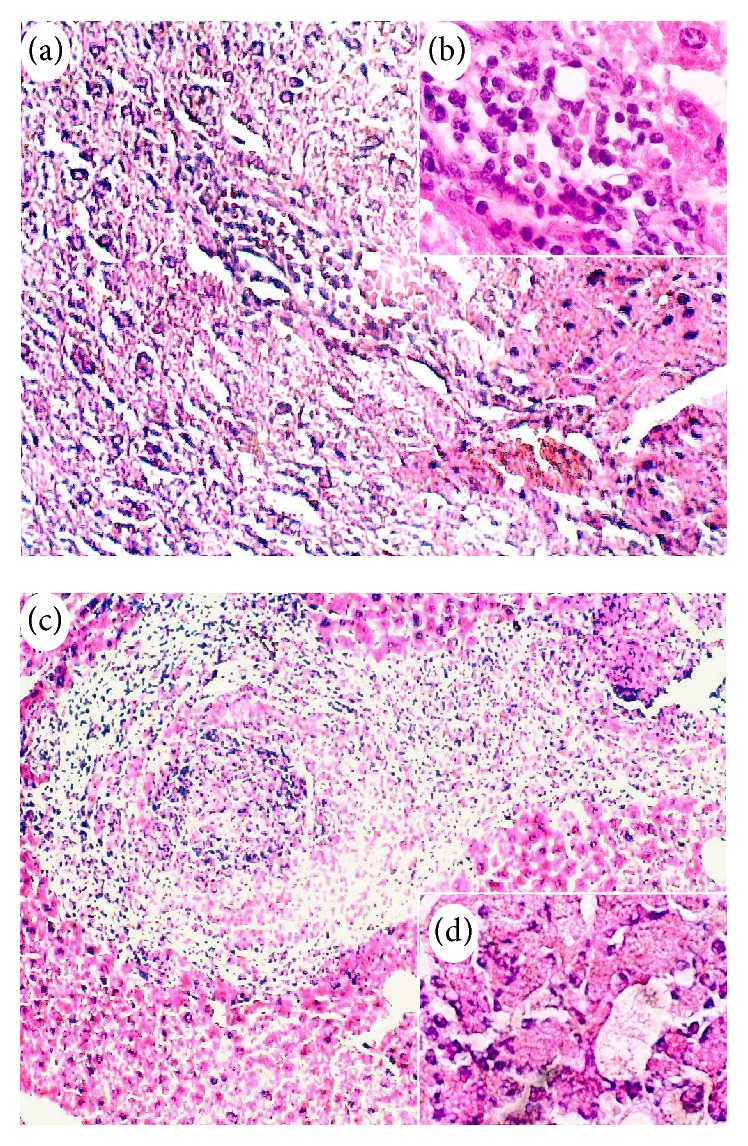
Effect of MPL treatment in ALA formation in BALB/c mice at four days postinfection. Histological changes in mice infected without MPL treatment. (a) Small foci of inflammatory infiltrate in the liver. Original magnification 10x. (b) Cellular infiltrate composed mainly by polymorphonuclear leukocytes. Original magnification 40x. Histological changes in infected mice and MPL-treated mice. (c) Granuloma with a necrotic center, inflammatory infiltrate, and trophozoites in the periphery. Original magnification 10x. (d) Cellular infiltrate near to trophozoites. Original magnification 40x. H&E stain.

Histological examination of livers from infected mice showed small foci of inflammatory infiltrate in some samples, mainly at 2 and 4 days p.i. (Figures 3(a) and 3(b), resp.); trophozoites were detected, although smaller than normal and with a circular morphology (Figure 2(a)).

Microscopic examination of lesions in infected and MPL-treated mice showed small necrosis areas accompanied by chronic granulomatous inflammation foci (Figures 3(c) and 3(d)). Cellular exudates included polymorphonuclear leukocytes and lymphocytes; multinucleated giant cells were observed in one preparation. Abundant trophozoites, with different morphology, were found at 2 days p.i. (Figure 2(b)), decreasing at 4 and 7 days p.i.

Moreover, in infected mice, trophozoites showed an apparent decrease in size in relation to that observed in trophozoites present in hamster. To analyze this, trophozoites were detected by IHC and a morphometrical analysis was performed to compare them with those trophozoites present in hamster liver lesions.

**Figure 4:**
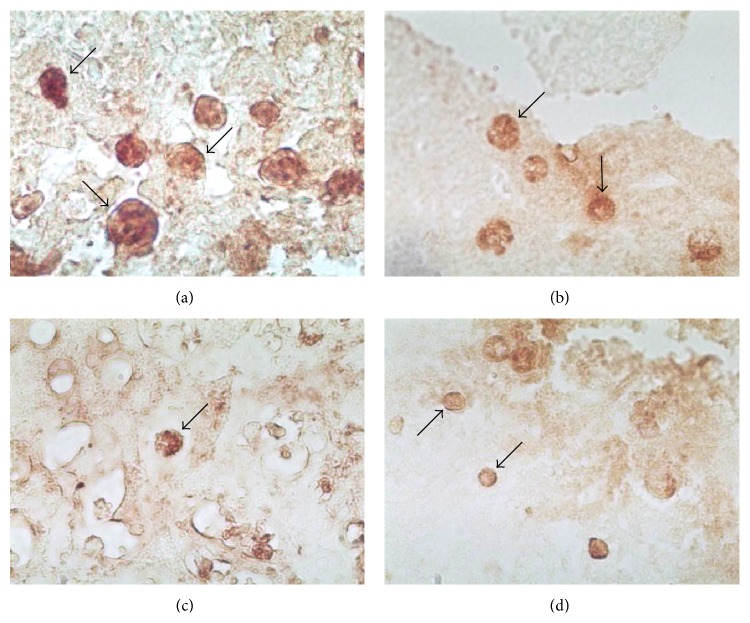
Morphological differences detected in trophozoites present in liver sections from hamster and BALB/c mice treated or not with MPL. Trophozoites were localized by IHC using a rabbit polyclonal antiserum against* E. histolytica*. (a) Trophozoites (arrows) present in liver tissue from infected hamster at four days p.i. Original magnification 40x. (b) Trophozoites present in liver from mice infected without MPL treatment at four days p.i. Original magnification 40x. (c) and (d) Trophozoites detected in liver from infected mice and MPL-treated mice at two and four days p.i., respectively. Original magnification 40x.

Trophozoites detected in liver from infected hamsters looked elongated with pseudopodia or rounded, and in most of the analyzed samples, peroxidase stain was strong and clearly defined (Figure 4(a)). In contrast, in nontreated infected mice, trophozoites were scarce and, at all times of postinfection tested, looked smaller and with a circular morphology (Figure 4(b)), while in MPL-treated and infected mice, standing trophozoites showed a diminution in size regardless of postinfection time (Figures 4(c) and 4(d)).

Results of morphometrical analysis of trophozoites found in the different experimental groups are described in Table 3. Trophozoites present in ALA from hamster, at different time p.i., did not have significant changes in major diameter (MD) or minor diameter (md). At two days postinfection a significant difference in the size was detected in trophozoites from mice with ALA compared with those detected in hamsters (Table 2, *P* < 0.01). However, no differences were observed in the size of trophozoites from infected mice treated or not with MPL (Table 2). Similar results were obtained at four and seven days p.i.; a significant diminution in the size of the trophozoites was observed in liver from mice, independently of the treatment, compared to trophozoites size from hamster.

Additionally, the size of the trophozoites present in liver from infected mouse treated or not with MPL showed a significant diminution in MD as well as in md (*P* < 0.01; Table 2) at 4 and 7 days p.i. in relation to the size observed in trophozoites found in samples from 2 days p.i. Trophozoites present in the livers from infected hamsters did not have significant differences in their diameters among postinfection times (*P* = 0.13 for MD and *P* = 0.32 for md).

## 4. Discussion

The mechanisms for murine host resistance to* E. histolytica* infection to develop hepatic abscesses are unknown. Some evidences with rabbit anti-mouse thymocyte globulin pretreated mice infected intracecally and intrahepatically suggest that cell mediated immunity plays a crucial protective role in this parasite infection [14]. However, studies realized by Stern et al. [4] demonstrated that resistance of nu/nu mice to infection only could be abolished with previous silica treatments, suggesting that macrophages provide the critical host defense in response to* E. histolytica*.

In our studies, despite the fact that damage percentage in MPL-treated mice was <15%, we observed that immune manipulation with MPL was able to induce an inflammatory process and hepatic features similar to those produced in susceptible animals. The mechanism by which MPL could participate in ALA development is unclear, but since MPL shares many effects on the immune system with PGE_1_, the mechanism could be explained as cytokine induced immunomodulation of macrophage function [9].

PGE_2_ can modulate immune responses preferentially towards TH2-cell subpopulations which inhibit macrophage activation by inhibition of IFN-*γ* production [5]. Previous work in hamsters has suggested that PGE_2_ plays an important role in immunomodulation of host's response in infection with this parasite, since* E. histolytica* infection in hamster increases plasmatic and local levels of PGE_2_ [6].

MPL shares with PGs the capability to regulate inflammatory cytokines and macrophage functions and may be an example of a natural feedback mechanism for controlling inflammation, which is consistent with the mechanism proposed by Bonta and Parnham [15]. Administration of MPL could be supplying the natural production of PGs in mice and modifying the adequate response against the amoeba.

In addition, the fact that MPL-treated mice did not develop bigger abscesses could be explained by the number of trophozoites used to inoculate mice, which was under the number usually used in resistant models. However, the participation of additional natural resistance mechanisms cannot be discarded.

Regarding morphological alterations in trophozoites, although diverse studies exist in which ALA development in susceptible and/or resistant models has been used, to date a study that describes the morphological changes that occur in trophozoites during the invasion has not been reported. Chavez-Munguia et al. described the ultrastructure of the trophozoites recovered from hamster ALA, using electronic microscopy [16]. They found a great diversity of sizes among recuperated trophozoites, which varies between 10 and 60 *μ*m. In our study, the MD of the trophozoites detected in hamsters had an average of 9.6 ± 2.7 *μ*m; nevertheless, we did not detect trophozoites with bigger dimensions in livers. Our observations suggest that trophozoites could modify their morphology as a consequence of microenvironment changes and tissue aggression by cells and/or components of the innate immune response of the host; differences between this study and that from Chavez-Munguia et al. could be due to the fact that, before their analysis, recovered trophozoites were transferred to axenic culture, which could support their growth and development. Moreover, data about trophozoites size in mice models are rather scarce. Cieslak et al., who obtained successful hepatic infection in SCID mice, reported trophozoites with a similar morphology as we described in this paper; however, authors did not discuss it [17]. Notwithstanding in our study, since a significant difference was not detected in the size of trophozoites present in mice treated with MPL in comparison to infected and untreated mice, this discarded a direct effect of MPL on parasites.

Concerning amoebic infection in BALB/c mice, although several studies have reported that, in resistant models, such as mice, trophozoites disappeared by 48 h p.i., we were able to detect trophozoites at longer times p.i., probably due to the use of IHC methodology; however, trophozoites were more abundant at 2 days p.i. decreasing by 4 and 7 days p.i., which agree with previously reported results [18, 19].

Finally, our results on infected and MPL-treated mice are consistent with Tsutsumi's discussion related to the proposal that the inflammatory process contributes to ALA formation.

Additional research could be focused to understand the role of an adequate control of the inflammatory process in the natural resistance to* E. histolytica* infection in BALB/c mice and its participation in regulation of the immune response.

## Figures and Tables

**Table 1:** Percentage of damage obtained in *E. histolytica* infected animals treated or not with MPL.

Specie	Treatment	Days p.i.	Weight (g)		Damage (%)
			Liver	Abscess	
Hamster	—	2	5.8 ± 1.3^*^	0.6 ± 0.1	10 ± 2
	—	4	6.5 ± 1.5^*^	1.3 ± 0.8	18 ± 7
	—	7	11.5 ± 4^*^	4.1 ± 0.4	35 ± 2

Mice	—	2	1.9 ± 0.4	0.227 ± 0.09	11 ± 5
	MPL	2	2.4 ± 0.4	0.215 ± 0.01	12 ± 3
	—	4	2.38 ± 0.9	0.236 ± 0.09	10 ± 2
	MPL	4	2.3 ± 0.4	0.273 ± 0.10	12 ± 2
	—	7	2.3 ± 0.4	0.191 ± 0.10	8 ± 0.1
	MPL	7	2.3 ± 0.2	0.192 ± 0.04	8 ± 0.1

^*^ *P* < 0.01 versus liver weight from noninfected hamsters.

**Table 2:** Frequencies of ALA presentation in *E. histolytica *infected BALB/c mice treated or not with MPL.

Group	Presence of ALA		
	2 days % (ratio)	4 days % (ratio)	7 days % (ratio)
Infected	36 (9/25)	50 (13/26)	61 (8/13)
Infected + MPL	76.9 (10/13)^*^	65 (13/23)^§^	60 (6/10)^#^

^*^ *P* = 0.03.

^§^ *P* = 0.37.

^#^ *P* = 0.63.

**Table 3:** Morphometric analysis of trophozoites detected in liver abscesses from experimental groups.

Days p.i.	BALB/c mice				Hamster	
	Infected		Infected + MPL		Infected	
	MD	md	MD	md	MD	md
**2**	5.8 ± 1.1^*^	4.9 ± 1.4	5.8 ± 1.4^*^	4.8 ± 1.3	9.6 ± 2.7	7.3 ± 2.1
**4**	4.1 ± 0.9^∗§*£*^	3.4 ± 0.8	3.0 ± 0.6^§*£*^	2.6 ± 0.60	8.9 ± 1.8	6.9 ± 0.62
**7**	2.7 ± 1.9^∗§*£*^	2.5 ± 0.5	2.1 ± 0.3^§*£*^	1.9 ± 0.28	8.8 ± 1.0	6.3 ± 1.26

MD: major diameter; md: minor diameter; MPL: misoprostol (10^−4^ M).

^*^ *P* < 0.01 difference among species.

^§^ *P* < 0.01 difference between treatments.

^*£*^ *P* < 0.01 difference between p.i. times.
